# Ileocecal valve syndrome and vitamin b12 deficiency after surgery: a multicentric prospective study

**DOI:** 10.1007/s13304-020-00845-z

**Published:** 2020-07-09

**Authors:** Paola Germani, Annalisa Zucca, Fabiola Giudici, Susanna Terranova, Marina Troian, Natasa Samardzic, Marco Greco, Jurij Janez, Camilla Gasparini, Emanuela Cagnazzo, Andrea Vignali, Fabio Giannone Codiglione, Andrea Armellini, Uberto Romario Fumagalli, Riccardo Rosati, Giuseppe Piccinni, Jacques Megevand, Ales Tomazic, Francesco Corcione, Silvia Palmisano, Nicolò de Manzini

**Affiliations:** 1 General Surgery Clinic, Department of Medical, Surgical and Health Sciences, University of Trieste, University Hospital of Trieste, Trieste, Italy; 2 General Surgery, Azienda Ospedaliera Dei Colli, Monaldi Hospital, Naples, Italy; 3 Department of Abdominal Surgery, Ljubljana University Medical Center, Ljubljana, Slovenia; 4 General Surgery, San Pio X Humanitas Research Hospital, Milan, Italy; 5 General Surgery, Santa Maria Hospital GVM Care and Research, Bari, Italy; 6 Department of Gastrointestinal Surgery, San Raffaele Hospital, Vita-Salute San Raffaele University, Milan, Italy; 7 General Surgery 2, Ospedali Civili, Brescia, Italy

**Keywords:** Ileocecal valve, Ileocecal junction, Vitamin B12, Quality of life, GIQLI, EORTC-QLQ-CR29

## Abstract

Patients undergoing colon resection are often concerned about their functional outcomes after surgery. The primary aim of this prospective, multicentric study was to assess the intestinal activity and health-related quality-of-life (HRQL) after ileocecal valve removal. The secondary aim was to evaluate any vitamin B12 deficiency. The study included patients undergoing right colectomy, extended right colectomy and ileocecal resection for either neoplastic or benign disease. Selected items of GIQLI and EORTC QLQ-CR29 questionnaires were used to investigate intestinal activity and HRQL before and after surgery. Blood samples for vitamin B12 level were collected before and during the follow-up period. The empirical rule effect size (ERES) method was used to explain the clinical effect of statistical results. Linear mixed effect (LME) model for longitudinal data was applied to detect the most important parameters affecting the total score. A total of 158 patients were considered. Applying the ERES method, the analysis of both questionnaires showed clinically and statistically significant improvement of HRQL at the end of the follow-up period. Applying the LME model, worsening of HRQL was correlated with female gender and ileum length when using GIQLI questionnaire, and with female gender, open approach, and advanced cancer stage when using the EORTC QLQ-CR29 questionnaire. No significant deficiency in vitamin B12 levels was observed regardless of the length of surgical specimen. In our series, no deterioration of HRQL and no vitamin B12 deficiency were found during the follow-up period. Nevertheless, warning patients about potential changes in bowel habits is mandatory. In our series, no deterioration of HRQL and no vitamin B12 deficiency were found during the follow-up period. Nevertheless, warning patients about potential changes in bowel habits is mandatory.

## Introduction

The ileocecal valve (ICV), also defined as ileocecal junction, is a sphincter valve that separates the small bowel from the large bowel, regulating the passage of the chymus under influence of hormones and nerve fibers [1, 2]. Removal of the ICV can lead to displacement of bacteria from the colon to the ileum and, under certain circumstances, it may result in a severe intestinal bacteria overgrowth (SIBO) syndrome, characterized by alteration in the number of bacteria in the upper gastrointestinal tract [3, 4]. The reported prevalence of SIBO in the general population is 0–20% and it increases up to 32% after resection of the ICV [5]. Symptoms are usually vague and non-specific, like abdominal discomfort, bloating, and diarrhea, but sometimes SIBO can lead to severe malabsorption, malnutrition, and vitamin B12 deficiency [4–8]. When considering vitamin B12 deficiency, it is important to note that, albeit this vitamin is actively absorbed exclusively in the terminal ileum, a small amount is passively absorbed throughout all the small bowel. Therefore, ileal resections shorter than 20 cm generally do not put patients at risk of developing vitamin B12 deficiency [9].

Since right colectomy and ileocecal resection are common surgical procedures performed for either malignant or benign diseases in both elective and emergency settings, patients often express their concern about potential postoperative functional outcomes. Although rarely, in the long-term period some patients may report a clinically relevant worsening in bowel habits, as well as vitamin deficiency and deterioration of quality of life, which can be cause for medico-legal issues. In this context, the primary aim of the present study was to evaluate the intestinal activity and quality of life of patients with uncomplicated postoperative course following right colectomy or ileocecal resection, whatever the primary indication for surgery had been. Secondly, postoperative vitamin B12 deficiency and possible supplementation requirements were assessed.

## Methods

This is a prospective, longitudinal, observational, multicentric study performed on patients referred to six Italian and one Slovenian colorectal surgery Centers between November 2016 and May 2018.

The study population included patients aged ≥ 16 years undergoing right colectomy, extended right colectomy, or ileocecal resection for either malignant or benign (i.e., inflammatory, ischemic) disease, in both elective and emergency settings. Surgical procedures were performed by open, laparoscopic (with either intra- or extra-corporeal anastomosis), or robotic approach. The following exclusion criteria were applied: palliative surgery, presence of diverting stoma, and postoperative complications grade ≥ 3b according to Clavien-Dindo classification [10].

The study was approved by all Centers (Protocol Number 5903, Local Ethical Committee of Trieste University Hospital) and written informed consent was obtained by all participants.

Operative data recorded for each patient included: age, gender, nature of disease (i.e., benign or malignant), surgical procedure, length of surgical specimen (i.e., total length, length of ileum, length of colon), cancer staging according to the AJCC classification [11] and adjuvant therapy in case of malignant disease.

Bowel function and quality of life before and after surgery were investigated by means of two validated questionnaires: the Gastrointestinal Quality of Life (GIQLI) questionnaire, which was administered to all patients with inflammatory, ischemic, or neoplastic disease, and the European Organization for Research and Treatment of Cancer (EORTC) QLQ-CR29 modules, which were selectively administered only to patients with neoplastic disease [12–14]. The GIQLI questionnaire was filled out by patients at the time of surgery, 2 weeks after surgery, and 6 weeks after surgery, evaluating the following selected items: abdominal pain, bloating, bowel frequency, bowel urgency, bowel movement, diarrhea, constipation, and nausea (i.e., question numbers 1, 3, 7, 30, 31, 32, 33, 36). A five-point scale was used to indicate how symptoms affected patient's quality of life (e.g., “0 = never”, “1 = rarely”, “2 = sometimes”, “3 = most of the time”, “4 = all the time”). Patients with neoplastic disease were also administered the EORTC QLQ-CR29 questionnaire at the time of surgery, 3 months after surgery, and 6 months after surgery, evaluating the following selected items: abdominal pain, bloating, gas and/or fecal incontinence, frequency of bowel movements during the day, and frequency of bowel movements during the night (i.e., question numbers 35, 37, 49, 50, 52, 53). A four-point scale was used to indicate how symptoms affected patient's quality of life (e.g., “1 = never”, “2 = occasionally”, “3 = most of the time”, “4 = all the time”) [12–16]. Chronic sequelae following surgery were defined as persistent symptoms determining a GIQLI score ≥ 3 at 6 weeks after surgery and/or a QLQ-CR29 score ≥ 3 at 6 months after surgery, respectively.

In addition, blood samples for vitamin B12 levels were collected at the time of surgery, 3 months after surgery and 6 months after surgery. Serum vitamin B12 levels between 148 pg/mL and 980 pg/mL were considered within normal range. Correlation between vitamin B12 levels and length of resected small bowel was evaluated.

### Statistical analysis

Data were prospectively collected through an anonymous database using Microsoft Excel 2007 (Microsoft Excel 2007, Redmond, WA, USA). Baseline characteristics were reported as mean ± standard deviation (SD) or median and interquartile range. Categorical variables were reported as frequency and percentage. Data regarding GIQLI and QLQ-CR29 score values at the time of surgery and over the course of follow-up were recorded as mean ± SD. Non-parametric Friedman test for paired data was used for analysis of variation in scores between baseline and follow-up. As post-hoc tests, pairwise comparisons were conducted using Wilcoxon signed rank test and corrected using the Holm method.

Statistically and clinically significant changes over time of questionnaire scores were evaluated. The severity of intervention-related symptoms was interpreted using minimal important difference (MID) determination evaluating a choice of specific items possibly influencing health-related quality-of-life (HRQL) outcomes (in addition to the clinical significance of the intervention itself) [16, 17]. To assess MID in measured results and explain their clinical effect, the empirical rule effect-size (ERES) calibration method was used, which defines a variation of 8% in the theoretical range of the same tool as a clinically significant modification in a HRQL outcome [18, 19]. In the present study, a variation of 8% in the score range (i.e., 0–4) of each single GIQLI item was equal to 0.32 points, whereas a variation of 8% in the score range (i.e., 1–4) of each QLQ-CR-29 item was equal to 0.24 points. In addition, linear mixed-effects (LME) model was applied for longitudinal data to detect the most important parameters (e.g., intraoperative, postoperative, and patient-related) affecting the total score.

All *p *values were measured from two-sided tests with 0.05 used as a significance level. All statistical analyses were conducted by R 3.5.0 software (R Foundation for Statistical Computing; https://www.r-project.org/) and STATA 14.2 (StataCorp, College Station, TX, USA).

## Results

The study analyzed a total of 158 patients undergoing right colectomy, right extended colectomy, or ileocecal resection between November 2016 and May 2018 at six Italian and one Slovenian colorectal surgery Centers. Of these, 87 (55.0%) patients were males and 71 (45.0%) patients were females. Median (range) age was 71 (16–91) years.

The main indication for surgery was malignant disease, which was reported in 149 (93.3%) patients (i.e., 148 colonic adenocarcinomas and 1 maltoma). According to AJCC classification, cancer patients were distributed as follows: 21 (14.2%) cancer stage 0, 29 (19.6%) cancer stage I, 52 (35.1%) cancer stage II, 40 (27.0%) cancer stage III, and 42 (2.7%) cancer stage IV. Cancer stage was not indicated in 2 (1.4%) patients. Overall, 46 (30.8%) patients underwent postoperative adjuvant chemotherapy.

Right colectomy was performed in 135 (85.4%) patients: of these, 127 (94.1%) patients presented with malignant disease and 8 (5.9%) patients presented with benign disease. Extended right colectomy was performed in 20 (12.7%) patients: all cases presented with malignant disease. Ileocecal resection was performed in 3 (1.9%) patients, two presenting with malignant disease and one presenting with benign disease. Laparoscopy was the preferred surgical approach, regarding 74.1% (117) of cases.

The median (range) length of the surgical specimen was 31 (16–152) cm. When analyzing the total length of the ileum and the colon, median (range) lengths for each segment were 8 (2–140) cm and 20 (4–55) cm, respectively. Table 1 summarizes the study population characteristics.

**Table 1:** Study population characteristics

Age, years	
Median (range)	71 (16–91)
Gender, *n* (%)	
Male	87 (55.0%)
Female	71 (45.0%)
Indication for surgery, *n* (%)	
Neoplastic disease	149 (93.3%)
Colonic adenocarcinoma	148 (99.3%)
Other	1 (0.7%)
Inflammatory/ischemic disease	9 (6.7%)
Type of surgery, *n* (%)	
Right colectomy	135 (85.4%)
Neoplastic disease	127 (94.1%)
Inflammatory/ischemic disease	8 (5.9%)
Right extended colectomy	20 (12.7%)
Neoplastic disease	20 (100.0%)
Inflammatory/ischemic disease	0 (0.0%)
Ileocecal resection	3 (1.9%)
Neoplastic disease	2 (66.7%)
Inflammatory/ischemic disease	1 (33.3%)
Surgical approach, *n* (%)	
Laparoscopic	117 (74.1%)
Open	40 (25.3%)
Robotic	1 (0.6%)
Specimen length, cm	
Median (range)	31 (16–152)
Ileum length, cm	
Median (range)	8 (2–140)
Colon length, cm	
Median (range)	20 (4–55)

Overall, the GIQLI questionnaire was filled out by 119 (75.3%) patients at baseline, 118 (74.7%) patients at 2 weeks after surgery, and 111 (70.3%) patients at 6 weeks after surgery, with a drop-out rate of 29.7%. Statistical analysis was performed only on patients completing the follow-up and a significant difference in symptoms between baseline and follow-up was recorded for all items, except for uncontrolled stools. Over the 6-week follow-up period, a clinically relevant improvement of all examined items was observed, except for diarrhea. Table 2 reports the GIQLI questionnaire scores over the study period.

**Table 2:** GIQLI questionnaire domains

Question	Baseline	2 weeks	6 weeks	*p *value
How often during the past 2 weeks have you had pain in the abdomen? (GIQLI #1)	1.73 ± 0.85	1.50 ± 0.68	1.35 ± 0.64^a^	< 0.001
How often during the past 2 weeks have you had bloating (sensation of too much gas in the abdomen)? (GIQLI #3)	2.02 ± 0.90	1.84 ± 0.63	1.34 ± 0.56^a^	< 0.001
How often during the past 2 weeks have you been troubled by frequent bowel movements? (GIQLI #7)	1.66 ± 0.77	1.75 ± 0.70	1.52 ± 0.67	< 0.001
How often during the past 2 weeks have you been troubled by urgent bowel movements? (GIQLI #30)	1.37 ± 0.71	1.58 ± 0.78	1.27 ± 0.66	< 0.001
How often during the past 2 weeks have you been troubled by diarrhea? (GIQLI #31)	1.39 ± 0.77	1.70 ± 0.77^a^	1.53 ± 0.72	< 0.001
How often during the past 2 weeks have you been troubled by constipation? (GIQLI #32)	1.98 ± 0.97	1.29 ± 0.69^a^	1.24 ± 0.61^a^	< 0.001
How often during the past 2 weeks have you been troubled by nausea? (GIQLI #33)	1.42 ± 0.79	1.25 ± 0.68	1.03 ± 0.46^a^	< 0.001
How often during the past 2 weeks have you been troubled by uncontrolled stools? (GIQLI #36)	1.02 ± 0.58	1.03 ± 0.51	0.98 ± 0.47	0.08
Total score	12.50 ± 4.16	12.10 ± 3.36	9.47 ± 3.70^a^	< 0.001

Score: “0 = never”, “1 = rarely”, “2 = sometimes”, “3 = most of the time”, “4 = all the time”

^a^Clinical significant changes according to the ERES (empirical rule effect-size) method

When considering chronic sequelae (i.e., GIQLI score ≥ 3 at 6 weeks after surgery), abdominal pain was reported by 2.7% of patients, bloating was reported by 1.8% of patients, frequent bowel movements were reported by 6.3% of cases, bowel urgency was reported by 5.4% of patients, diarrhea was reported by 7.2% of patients, constipation was reported by 3.6% of patients, nausea was reported by 0.9% of patients, and uncontrolled stools were reported by 0.9% of patients (Table 3).

**Table 3:** Chronic sequelae (GIQLI score ≥ 3) at 6 weeks after surgery

GIQLI item	Frequency, *n* (%)
Abdominal pain	3 (2.7%)
Bloating (sensation of too much gas in the abdomen)	2 (1.8%)
Frequent bowel movements	7 (6.3%)
Urgent bowel movements	6 (5.4%)
Diarrhea	8 (7.2%)
Constipation	4 (3.6%)
Nausea	1 (0.9%)
Uncontrolled stools	1 (0.9%)

**Fig. 1:**
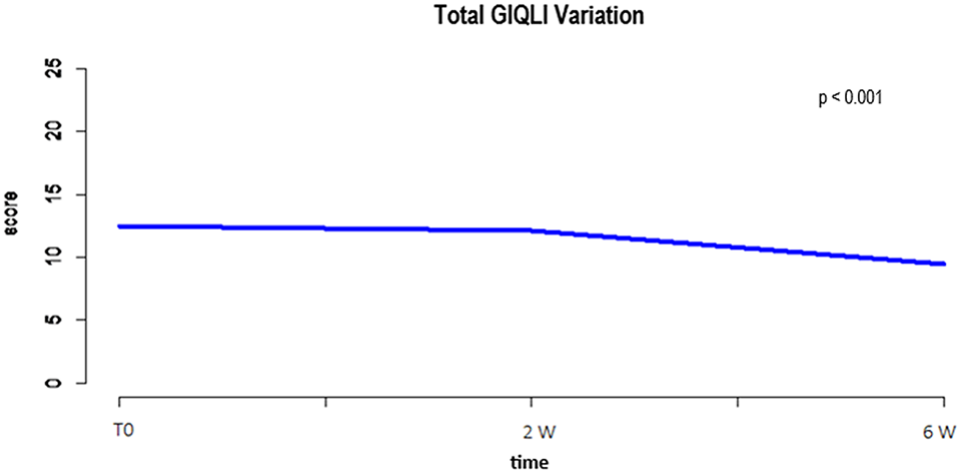
Total GIQLI variations

Applying the ERES method to the GIQLI questionnaire scores, the analysis showed that constipation significantly improved 2 weeks after surgery, maintaining its improvement at the 6-week follow-up, whereas diarrhea was found to significantly worsen 2 weeks after surgery before improving again at 6 weeks after surgery. Abdominal pain, bloating and nausea were found to significantly improve over the 6-week follow-up period (*p* < 0.001). Results are summarized in Table 2 and Fig. 1.

**Fig. 2:**
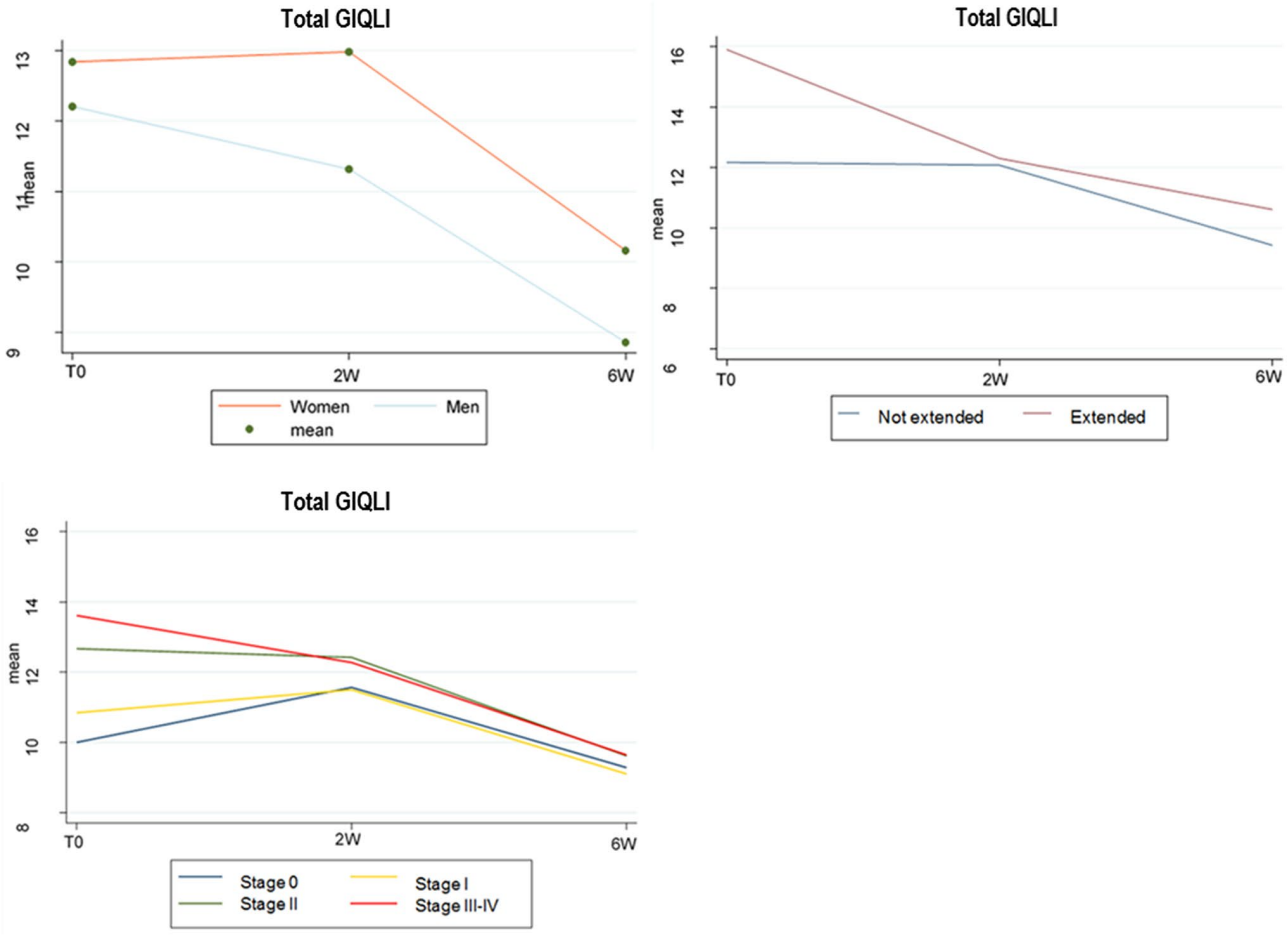
Statistically significant data for global worsening of GIQLI score

Applying LME model for longitudinal data, the statistically significant correlation between clinical parameters and worsening of symptoms at 6 weeks after surgery is reported in Table 4 and Fig. 2. Specifically, female gender was found to affect the frequency of abdominal pain, bloating, frequent bowel movements, diarrhea, and nausea. Advanced age and open approach were correlated with constipation. Advanced AJCC stage at the time of surgery was related to postoperative abdominal pain, bloating, frequent bowel movements, and urgent bowel movements. The specimen length correlated with bloating, frequent bowel movements, and urgent bowel movements. When considering the GIQLI total score at 6 weeks after surgery, worsening of HRQL was found to be significantly correlated with female gender (*p* = 0.03) and length of resected ileum (*p* = 0.04).

**Table 4:** Correlation between clinical parameters and worsening GIQLI scores at 6 weeks after surgery

GIQLI	1		3		7		30		31		32		33		36		Total	
	*β*	*p*	*β*	*p*	*β*	*p*	*β*	*p*	*β*	*p*	*β*	*p*	*β*	*p*	*β*	*p*	*β*	*p*
Age	− 0.005	0.17	− 0.001	0.71	0.002	0.55	0.006	0.07	0.006	0.32	0.001	0.005	− 0.002	0.45	0.0002	0.93	0.02	0.29
Gender																		
Female	Ref	0.02	Ref	0.03	Ref	0.02	Ref	0.06	Ref	0.01	Ref	0.99	Ref	0.01	Ref	0.5	Ref	0.03
Male	− 0.22		− 0.21		− 0.26		− 0.19		− 0.25		0.001		− 0.24		− 0.05		− 1.2	
Surgical approach																		
Laparoscopic	Ref	0.32	Ref	0.23	Ref	0.83	Ref	0.06	Ref	0.54	Ref	0.03	Ref	0.9	Ref	0.07	Ref	0.24
Open	0.118		− 0.13		− 0.03		0.21		0.07		0.26		0.01		0.15		0.73	
Type of surgery																		
Right colectomy	Ref	0.08	Ref	0.03	Ref	0.04	Ref	0.08	Ref	0.12	Ref	0.32	Ref	0.02	Ref	0.83	Ref	0.08
Ext.right colectomy	0.32		0.37		0.4		0.32		0.28		0.99		0.4		0.22		1.72	
Ileocecal resection																		
Ileum length	0.001	0.69	0.007	0.05	0.005	0.19	0.001	0.69	0.007	0.04	− 0.001	0.79	0.007	0.03	0.004	0.13	0.04	0.04
Specimen length	0.005	0.28	0.005	0.21	0.006	0.19	0.009	0.03	0.004	0.32	− 0.002	0.58	0.002	0.54	0.002	0.45	0.036	0.11
Colon length	0.004	0.42	− 0.007	0.21	− 0.001	0.8	0.01	0.02	− 0.009	0.08	− 0.002	0.75	− 0.01	0.01	− 0.005	0.2	− 0.029	0.34
Adjuvant therapy																		
No	Ref	0.25	Ref	0.17	Ref	0.9	Ref	0.38	Ref	0.11	Ref	0.48	Ref	0.23	Ref	0.12	Ref	0.66
Yes	0.13		0.14		− 0.01		− 0.1		− 0.17		− 0.08		− 0.12		− 0.13		− 0.26	
AJCC stage																		
0	Ref		Ref		Ref		Ref		Ref		Ref		Ref		Ref		Ref	
I	0.382	0.035	0.131	0.47	0.221	0.27	0.318	0.1	− 0.128	0.49	− 0.048	0.81	− 0.183	0.31	− 0.014	0.93	0.1976	0.86
II	0.379	0.018	0.206	0.19	0.418	0.02	0.539	0.001	0.007	0.96	0.1152	0.52	− 0.129	0.41	− 0.087	0.51	1.289	0.17
III–IV	0.64	0.001	0.378	0.02	0.407	0.02	0.394	0.02	− 0.192	0.25	0.261	0.14	− 0.05	0.75	− 0.084	0.72	1.557	0.1

Overall, the EORTC QLQ-CR29 questionnaire was filled out by 148 (93.7%) patients at baseline, 135 (85.4%) patients at 3 months after surgery, and 102 (64.5%) patients at 6 months after surgery, with a drop-out rate of 35.4%. Statistical analysis was performed only on patients completing the follow-up and a significant improvement in symptoms between baseline and follow-up were recorded for all items, except for gas and fecal incontinence. Table 5 reports the EORTC QLQ-CR29 questionnaire scores over the study period.

**Table 5:** EORTC QLQ-CR29 questionnaire domains

Question	Baseline	3 months	6 months	*p* value
Did you have abdominal pain? (QLQ-CR29 #35)	1.73 ± 0.85	1.50 ± 0.68^a^	1.35 ± 0.64^a^	< 0.001
Did you have a bloated feeling in your abdomen? (QLQ-CR29 #37)	2.04 ± 0.87	1.64 ± 0.79^a^	1.47 ± 0.71^a^	< 0.001
Have you had unintentional gas release/flatulence? (QLQ-CR29 #49)	1.49 ± 0.83	1.49 ± 0.81	1.49 ± 0.78	0.17
Have you had any stool leakage from your back passage? (QLQ-CR29 #50)	1.16 ± 0.54	1.14 ± 0.52	1.17 ± 0.51	0.63
Did frequent bowel movements occur during the day? (QLQ-CR29 #52)	1.88 ± 0.89	1.76 ± 0.83	1.57 ± 0.70^a^	< 0.001
Did frequent bowel movements occur during the night? (QLQ-CR29 #53)	1.44 ± 0.69	1.27 ± 0.62	1.22 ± 0.52	< 0.001
Total score	9.73 ± 3.05	8.80 ± 2.88	8.28 ± 2.65	< 0.001

Score: “1 = never”, “2 = occasionally”, “3 = most of the time”, “4 = all the time”

^a^Clinical significant changes according to the ERES (empirical rule effect-size) method

When considering chronic sequelae (i.e., EORTC QLQ-CR29 score ≥ 3 at 6 months after surgery), abdominal pain was reported by 4.9% of patients, bloating was reported by 8.8% of patients, unintentional release of gas was reported by 12.7% of patients, stool leakage was reported by 3.9% of patients, and frequent bowel movements were reported by 7.8% of patients during the day and 2.9% of patients during the night, respectively (Table 6).

**Table 6:** Chronic sequelae (EORTC QLQ-CR29 score ≥ 3) at 6 months after surgery

GIQLI item	Frequency, *n* (%)
Abdominal pain	5 (4.9%)
Bloating (sensation of too much gas in the abdomen)	9 (8.8%)
Unintentional gas release/flatulence	13 (12.7%)
Stool leakage	4 (3.9%)
Frequent bowel movements during the day	8 (7.8%)
Frequent bowel movements during the night	3 (2.9%)

**Fig. 3:**
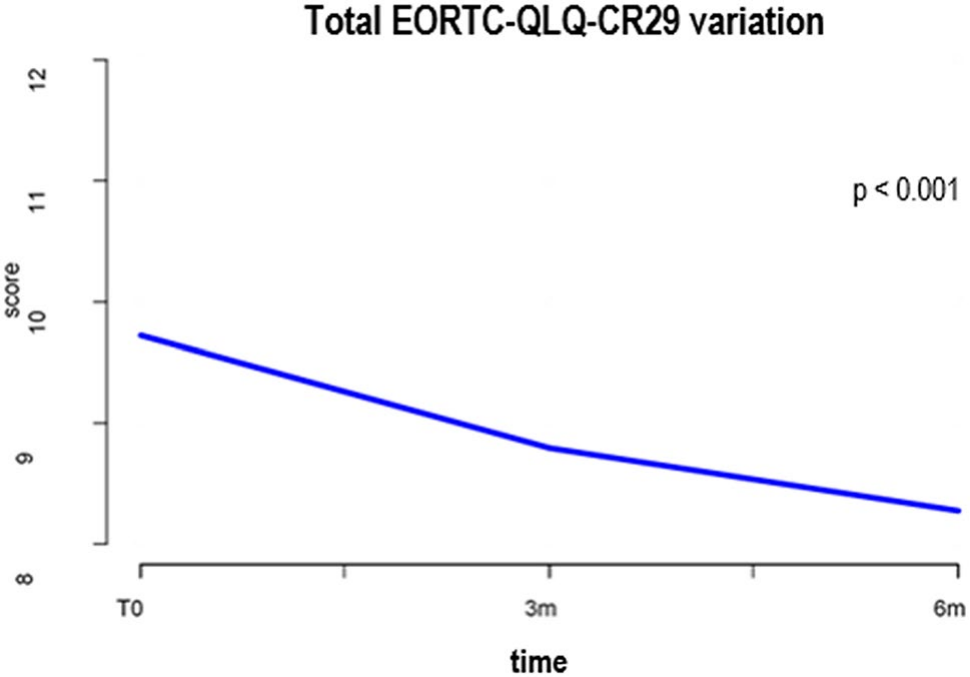
Total EORTC QLQ-CR29 variations

Applying the ERES method to the EORTC QLQ-CR29 questionnaire scores, the analysis showed a significant improvement of reported abdominal pain, bloating, and frequency of bowel movements during the day at 6 months after surgery. Overall, the total score was significantly improved during the 6-month follow-up period (*p* < 0.001). Results are summarized in Table 5 and Fig. 3.

**Fig. 4:**
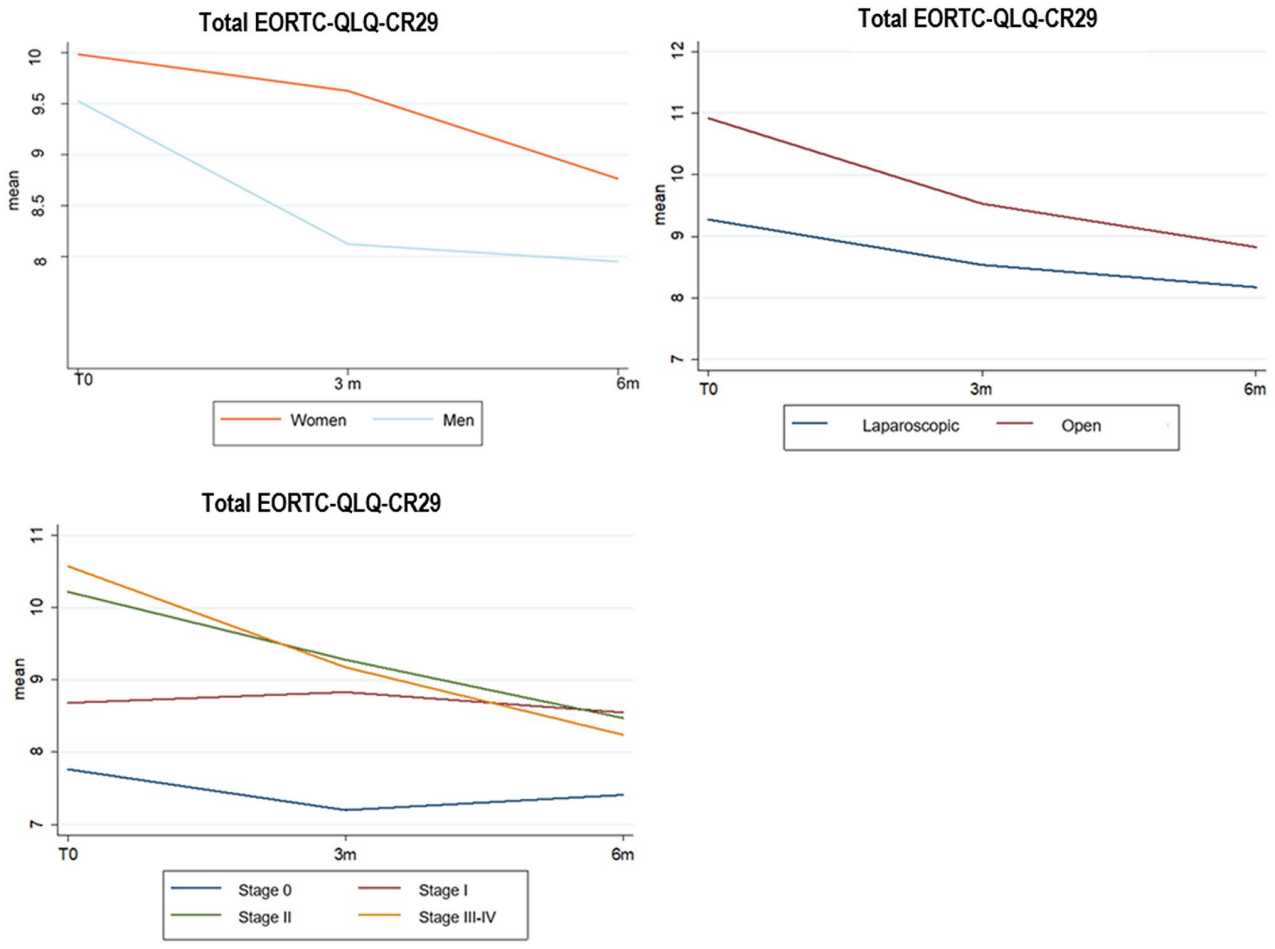
statistically significant data for global worsening of EORTC QLQ-CR29 score

Applying LME model for longitudinal data, the statistically significant correlation between clinical parameters and worsening of symptoms at 6 months after surgery is reported in Table 7 and Fig. 4. Specifically, female gender was found to affect the frequency of bowel movements. Advanced age was linked to stool leakage. Open approach was correlated to postoperative abdominal pain, gas incontinence, and stool leakage. Advanced AJCC stage at the time of surgery was related to abdominal pain, bloating, and frequent bowel movements during the day. The specimen length correlated with gas incontinence, stool leakage, and frequent bowel movements during the day. When considering the EORTC QLQ-CR29 total score at 6 months after surgery, worsening of HRQL was found to be significantly correlated with female gender (*p* = 0.02), open approach (*p* = 0.0017), and advanced cancer stage (*p* = 0.005).

**Table 7:** Correlation between clinical parameters and worsening EORTC QLQ-CR29 scores at 6 months after surgery

EORTC QLQ-CR29	35		37		49		50		52		53		Total	
	*β*	*p*	*β*	*p*	*β*	*p*	*β*	*p*	*β*	*p*	*β*	*p*	*β*	*p*
Age	− 0.003	0.38	− 0.0007	0.84	0.006	0.11	0.006	**0.011**	− 0.001	0.78	0.004	0.15	0.01	0.4
Gender														
Female	Ref	0.4	Ref	0.07	Ref	0.4	Ref	0.2	Ref	0.002	Ref	0.18	Ref	0.02
Male	− 0.08		− 0.19		− 0.099		− 0.089		− 0.323		− 0.106		− 0.883	
Surgical approach														
Laparoscopic	Ref	0.03	Ref	0.83	Ref	0.004	Ref	0.003	Ref	0.69	Ref	0.12	Ref	0.017
Open	0.25		0.03		0.39		0.236		0.05		0.12		1.096	
Type of surgery														
Right colectomy	Ref	0.96	Ref	0.59	Ref	0.0058	Ref	0.97	Ref	0.05	Ref	0.07	Ref	0.82
Ext.right colectomy	0.007		0.09		− 0.48		− 0.035		1.9		− 0.212		0.134	
Ileocecal resection														
Ileum length	0.005	0.2	− 0.0007	0.86	− 0.009	0.04	− 0.003	0.26	− 0.0005	0.9	− 0.0028	0.34	− 0.01	0.45
Specimen length	0.004	0.32	0.002	0.59	− 0.0018	0.72	0.001	0.73	0.001	0.81	0.0005	0.86	0.0074	0.66
Colon length	− 0.003	0.6	0.004	0.37	0.014	0.0192	0.007	0.046	0.003	0.64	0.006	0.13	0.032	0.11
Adjuvant therapy														
No	Ref	0.34	Ref	0.79	Ref	0.029	Ref	0.03	Ref	0.52	Ref	0.46	Ref	0.39
Yes	0.11		− 0.03		− 0.28		− 0.161		0.076		− 0.06		− 0.369	
AJCC stage														
0	Ref		Ref		Ref		Ref		Ref		Ref		Ref	
I	0.041	0.81	0.372	0.0462	0.201	0.33	0.131	0.31	0.269	0.15	0.163	0.24	1.182	0.09
II	0.381	0.01	0.318	0.0533	0.36	0.05	0.157	0.16	0.479	0.004	0.202	0.1	1.884	0.002
III− IV	0.393	0.01	0.342	0.0455	0.209	0.27	0.101	0.39	0.548	0.002	0.198	0.12	1.767	0.005

When considering whether patients underwent cholecystectomy before, during, or after primary surgery, data were available for 31 patients. Specifically, cholecystectomy was performed before colic resection in 1 patient (i.e., 5 years before surgery), during primary surgery in 3 patients, and after primary surgery in 27 patients. Of these, no one underwent cholecystectomy within the first 6 months after surgery.

Regarding the serum levels of vitamin B12, blood sampling was performed in 158 (100.0%) patients at baseline, 133 (84.2%) patients at 3 months after surgery, and 92 (58.2%) patients at 6 months after surgery. Statistical analysis was performed only on patients completing the 6-month follow-up. Baseline median (range) value was 307.0 (81.9–2295.2) pg/mL. Median (range) values at 3 and 6 months after surgery were 287 (76.0–1394.0) pg/mL and 294.0 (73.8–892.3) pg/mL, respectively. Longitudinal analysis generally showed a significant decrease over time in vitamin B12 median (range) levels [i.e., 307.0 (81.9–2295.2) pg/mL at baseline, 287.0 (76.0–1394.0) pg/mL at 3 months, and 294.0 (73.8–892.3) pg/mL at 6 months, respectively; *p* < 0.001]. When considering data accordingly to the length of resected ileum, patients with resected ileum length ≥ 20 cm still presented a significant decrease in vitamin B12 median (range) levels, already evident at 3 months after surgery [i.e., 376.0 (185.0–631.0) pg/mL at baseline, 372.0 (207.0–596.0) pg/mL at 3 months, and 325.0 (131.0–564.0) pg/mL at 6 months, respectively; *p* < 0.001]. On the contrary, patients with resected ileum length < 20 cm, presented an increase in vitamin B12 median (range) levels that was statistically significant at 6 months after surgery compared to baseline values [i.e., 240.0 (81.9–2295.2) pg/mL at baseline, 255.5 (76.0–1394.0) pg/mL at 3 months, and 265.0 (73.8–892.3) pg/mL at 6 months, respectively; *p* = 0.02]. Data are summarized in Table 8.

**Table 8:** Vitamin B12 levels during 6-month follow-up according to resected ileum length

	Baseline vitamin B12 median (range) value, pg/mL	3-month vitamin B12 median (range) value, pg/mL	6-month vitamin B12 median (range) value, pg/mL	*p* value
Any ileum length (*n* = 92)	307.0 (81.9–2295.2)	287.0 (76.0–1394.0)	294.0 (73.8–892.3)	< 0.001
Resected ileum length < 20 cm (*n* = 59)	240.0 (81.9–2295.2)	255.5 (76.0–1394.0)	265.0 (73.8–892.3)	0.02
Resected ileum length ≥ 20 cm (*n* = 33)	376.0 (185.0–631.0)	372.0 (207.0–596.0)	325.0 (131.0–564.0)	< 0.001

## Conclusions and discussion

After ICV removal, patients can experience changes in bowel habits, that vary in a vast array of clinical manifestations and may be correlated with small bowel bacteria overgrowth (SIBO) [20]. Although in the majority of cases bowel function after ICV removal is reported as satisfactory [16], a significant postoperative dysfunction may sometimes ensue, affecting general well-being and patient HRQL to the point that it may become a potential cause for medico-legal issues. As outlined by the Italian Society of Legal Medicine and Insurance Policy (SIMLA), according to the Italian legislation, alterations of quality of life after intestinal resection can justify the patients claim for disability compensation. The presumptive damage is assessed by means of a score that takes into consideration the length of bowel resection, the seriousness of clinical signs and symptoms, the subsequent need for further medical treatment, and the postoperative weight loss [21–23].

In the present study, we evaluated postoperative quality of life of patients undergoing ICV removal surgery by means of two validated questionnaires. Specifically, data from the GIQLI questionnaire showed a progressive significant clinical improvement over the 6-week follow-up period for all reported symptoms, except for uncontrolled stools. Reported diarrhea was found to worsen at 2-week follow-up before improving 4 weeks thereafter. The initial worsening of diarrhea could be explained by the lack of water absorption after right colon removal. However, the transiency of the symptom is probably related to compensatory mechanisms in the remnant colon. Overall, the quality of life as expressed by GIQLI total score was found to improve significantly throughout the follow-up period.

When analyzing data from the EORTC QLQ-CR29 questionnaire, a similar progressive significant clinical improvement was observed over the 6-month follow-up period for all reported symptoms, except for gas and fecal incontinence. Overall, the quality of life as expressed by the total score was found to improve significantly throughout the follow-up period. When applying the ERES method, we found a significant amelioration of abdominal pain, bloating, and frequency of bowel movements during the day over the 6-month follow-up period. This is in accordance with Theodoropoulous et al., who reported that “defecation problems” after laparoscopic colectomy for cancer worsen 2–3 weeks after surgery and progressively return to baseline values 3 months after surgery [24–26].

In the present study, open approach, extended right colectomy, and specimen length were responsible for postoperative worsening of clinical symptoms. In addition, an advanced AJCC stage was significantly correlated with higher score rates for abdominal pain, bloating, and frequency of bowel movements. This is in accordance with literature data [25] and it is probably related to the fact that advanced stages of disease may usually require extended surgery and adjuvant therapies.

As expected, constipation and stool leakage worsened with age and this is probably related to other factors influencing bowel movements and sphincter activity. Indeed, several studies report that constipation prevalence worldwide varies between 2 and 20%, but it increases up to 33.5% in adults aged 60 years or older, especially women [27, 28].

When considering fecal incontinence, the condition was not impaired by any of the considered parameters at 6 weeks after surgery. This can be easily explained considering that the surgical procedures performed (i.e., right colectomy, extended right colectomy, and ileocecal resection) do not involve dissection of the inferior hypogastric plexus or the anal sphincter complex. Somewhat unsurprisingly, when further analyzing the prevalence of reported fecal and gas incontinence at 6 months after surgery, we found that all patients complaining the symptoms were undergoing adjuvant chemotherapy. Therefore, it is fair to suppose that therapy-related effects, more than the surgical procedure per se, are to be related to symptoms of bowel incontinence.

In the present study, the main parameters correlated with worse HRQL scores at 6 weeks after surgery were female gender and length of resected ileum, whereas at 6-month follow-up the involved factors were female gender, open approach, and advanced cancer stage. These results can be explained when considering that, in the first postoperative period, bowel function may be influenced by the length of resected bowel but subsequently the remnant intestine adjusts to compensate for absorptive functions. Later, bowel habits are more easily related to the advanced stage of disease, the need for further medical treatment, and, in case of open approach, the formation of adhesions that may hinder normal bowel motility. When considering the gender influence on reported bowel habits, our results are in accordance with literature data. In fact, several studies have reported a post-surgical worsening of gastrointestinal function [24, 25, 29]. In particular, Theodoropoulos et al. demonstrated that there is a higher frequency of reported postoperative bloating and urgency in female patients compared to male patients after gastrointestinal surgery [24, 25].

Overall, reported defecation problems persisting 6 months after surgery ranged between 2.9% and 12.7%, which is generally lower than what reported by literature data (15–45%) [29, 30]. However, no significant HRQL impairment and disabling chronic sequelae were found after right colectomy, right extended colectomy, and ileocecal resection, and these results are in accordance with literature studies reporting usually satisfactory bowel function and overall quality of life comparable to general population after segmental colectomy for cancer [16, 24–26, 29, 30].

As for vitamin deficiencies, it is well known that extended small bowel resection (length > 20 cm) can lead to malabsorption [9] and restorative proctocolectomy with ileal pouch can result in vitamin deficiency due to enteral stasis and subsequent higher risk of SIBO [31]. However, vitamin B12 deficiency after ICV removal is scarcely documented in literature. To our knowledge, this is one of the first studies investigating vitamin B12 deficiency after right colectomy, extended right colectomy, and ileocecal resection. Although results are preliminary, in the present study we demonstrated a general significant decrease in vitamin B12 levels, which was evident already at 3 months after surgery although median values always remained within normal laboratory range. Moreover, when analyzing vitamin B12 levels according to the length of resected ileum, we found a statistically significant decrease over time in median vitamin B12 levels in patients with resected ileum length ≥ 20 cm. On the contrary, patients with resected ileum length < 20 cm presented a significant increase in vitamin B12 at 6 months after surgery compared to basal and 3 months values. These results can be explained considering that vitamin B12 absorption primarily takes place in the distal ileum, although it is passively absorbed throughout all the small bowel [9]. Possible adaptive mechanisms may intervene to guarantee an adequate absorption of vitamin B12. However, considering the limited sample size and the fact the median values remained within normal laboratory range, the surgical procedures examined may not be blamed per se on the potential postoperative vitamin deficiency, but definite conclusions require further studies.

Despite its prospective design, the present study has several limitations. Firstly, the small sample size may prevent adequate generalization of results. In addition, the investigated items are extremely subjective and influenced by several factors that may not have been adequately considered despite an appropriate statistical analysis (e.g., the possible influence of previous, concurrent, or subsequent cholecystectomy [32, 33]). Finally, a relatively high drop-out rate was recorded in both the compilation of questionnaires and the dosage of vitamin B12, thus potentially hindering a complete rationalization of results. Nevertheless, we tried to overcome these limitations using appropriate statistical analyses (i.e., ERES method and LME model for longitudinal data), to assess the clinical significance of each considered item and to detect the most important parameters potentially affecting HRQL outcomes. In this context, the strength of the study relies on its prospective, multicentric design and the fact that subjective parameters were investigated by means of two well-validated questionnaires.

In conclusion, in the present series right colectomy, extended right colectomy, and ileocolic resection were not associated with significant deterioration in patient’s quality of life nor with significant vitamin B12 deficiency up to 6 months after surgery. However, it appears to be mandatory to give patients adequate preoperative counseling so as to warn about potential changes in bowel habits and functions. Patients need to be informed that bowel changes can occur, although not very frequently and generally transient in nature. In this context, any malpractice allegations and claim for disability compensation should be considered mostly inappropriate and carefully evaluated.
