# Correction to: Ileocecal valve syndrome and vitamin b12 deficiency after surgery: a multicentric prospective study

**DOI:** 10.1007/s13304-020-00851-1

**Published:** 2020-08-01

**Authors:** Paola Germani, Annalisa Zucca, Fabiola Giudici, Susanna Terranova, Marina Troian, Natasa Samardzic, Marco Greco, Jurij Janez, Camilla Gasparini, Emanuela Cagnazzo, Andrea Vignali, Fabio Giannone Codiglione, Andrea Armellini, Uberto Romario Fumagalli, Riccardo Rosati, Giuseppe Piccinni, Jacques Megevand, Ales Tomazic, Francesco Corcione, Silvia Palmisano, Nicolò de Manzini

**Affiliations:** 1 General Surgery Clinic, Department of Medical, Surgical and Health Sciences, University of Trieste, University Hospital of Trieste, Trieste, Italy; 2 General Surgery, Azienda Ospedaliera Dei Colli, Monaldi Hospital, Naples, Italy; 3 Department of Abdominal Surgery, Ljubljana University Medical Center, Ljubljana, Slovenia; 4 General Surgery, San Pio X Humanitas Research Hospital, Milan, Italy; 5 General Surgery, Santa Maria Hospital GVM Care and Research, Bari, Italy; 6 Department of Gastrointestinal Surgery, San Raffaele Hospital, Vita-Salute San Raffaele University, Milan, Italy; 7 General Surgery 2, Ospedali Civili, Brescia, Italy

## Correction to: Updates in Surgery 10.1007/s13304-020-00845-z

One of the co-author Eliana Cagnazzo has been incorrectly published. The correct co-author name has been copied below:

Emanuela Cagnazzo

The original article has been corrected.

